# Adjusting sowing date to enhance roselle performance and water productivity under water deficiency stress

**DOI:** 10.1186/s12870-026-09455-0

**Published:** 2026-07-16

**Authors:** Mohamed A. A. Ahmed, Alia Amer, S. M. Abolmaaty, Karam A. Elzopy, Ekramy Abdel Moatamed Atef, Engy A. Sultan

**Affiliations:** 1 Plant Production Department (Horticulture - Medicinal and Aromatic Plants), Faculty of Agriculture (Saba Basha), Alexandria University, Alexandria, 21531 Egypt; 2 Medicinal and Aromatic Plants Research Department, Horticulture Research Institute, Agricultural Research Center, Giza, Egypt; 3 Central Laboratory for Agricultural Climate, Agricultural Research Centre, Cairo, Egypt; 4 Department of Soil and Agricultural Chemistry, Faculty of Agriculture, Saba Basha, Alexandria University, Alexandria, Egypt

**Keywords:** Anthocyanin, Roselle, Planting dates, Productivity, Water saving

## Abstract

Climate change induced water scarcity poses a critical challenge to sustainable crop production and an escalating threat to food security, particularly in arid and semi-arid regions Adjusting sowing dates in combination with deficit irrigation to enhance water productivity is a successful adaptation strategy to mitigate the effects of water scarcity on crop production. However, limited information is available on the interactive effects of sowing timing and irrigation levels on roselle (*Hibiscus sabdariffa* L.) under arid and semi-arid conditions. In this context, a field experiment was conducted during 2021 and 2022 seasons at the AL-Busili Experimental Farm of the Central Laboratory for Agricultural Climate, Agricultural Research Center in Egypt, to assess the effects of three sowing dates (T1: May 19, T2: June 19, and T3: July 19) and three irrigation regimes (I1: 100%, I2: 75%, and I3: 50% of crop evapotranspiration “ETc”) on growth performance, yield and water productivity of roselle. The treatments were arranged in a split-plot design with three replications. The results indicated that sowing on June 19 (T2) under full irrigation (100% ETc) significantly increased plant height (253.17 cm), branch number (25.74 plant^− 1^), fruit number (100.30 plant^− 1^), and sepal dry weight (18.32 g plant^− 1^). Deficit irrigation (75% and 50% ETc) enhanced anthocyanin accumulation, with the highest value (52.69 mg g^− 1^ DW) recorded under 75% ETc. Notably, the combination of late sowing (July 19) and severe water deficit (50% ETc) achieved the uppermost water productivity, reaching 1.917 and 1.922 kg m^− 3^ in the first and second seasons, respectively, despite a reduction in yield. Based on these findings, it could be concluded that the late sowing date can be a viable management strategy in Egypt with limited water availability in terms of water productivity of harvested roselle fruits. These findings highlight a critical comparison between maximizing yield and improving water productivity, suggesting that adjusting sowing date in integration with deficit irrigation can serve as a successful climate adaptation strategy for roselle cultivation in water-limited environments.

## Introduction

Roselle (*Hibiscus sabdariffa L.*) is an annual summer plant from the family Malvaceae, commonly referred to as “Karkade” in most Arab countries, including Egypt [1]. Egypt is considered the country where roselle originated [2]. It is one of the herbal drugs; it is rich in vitamin C, organic acids (tartaric, citric, malic, and oxalic acids), and two types of anthocyanin, namely gossypetin (cyanidin) and hibiscin (delphinidin) [3]. Numerous environmental agronomic parameters impact roselle plant growth, yield, and quality [4]. The biggest challenge to obtaining good agricultural production globally is drought stress. Moreover, increasing crop yields and conserving irrigation water are two connected and significant worldwide challenges. A few efficient methods for using scarce water resources are choosing the right planting date and managing irrigation [5].

Roselle is generally considered a moderately drought-tolerant crop that can maintain growth and productivity under moderate water-deficit conditions through the activation of several physiological and biochemical adaptation mechanisms. These adaptive responses may include osmotic adjustment, regulation of photosynthetic activity, maintenance of nutrient uptake, and enhanced accumulation of protective secondary metabolites such as anthocyanins and phenolic compounds. Such mechanisms enable roselle plants to partially mitigate the adverse effects of drought stress and sustain metabolic activity under limited water availability. However, prolonged or severe water deficits may still negatively affect growth, yield, and overall plant performance. Therefore, understanding the interaction between irrigation management and planting date is essential for optimizing roselle productivity under water-limited environments [6, 7].

Agronomic measures like sowing timing enhance high yield by promoting plant development and growth, improving land economics, and ensuring the plant’s vulnerable growth stage aligns with environmental conditions [6]. Roselle is a short-day plant that needs a photoperiod of 12 to 12.5 h to bloom. Long days during the wrong stage of development lead to yield loss [7]. The sown roselle’s relative earliness is caused by photosensitivity and temperature. Such plants might bloom early under conditions of short levels of sunlight and may not bloom when the sunlight interval exceeds 11 h [8]. *H. sabdariffa* L. plants, which were sown on 15th April, had significant increases in all growth characteristics [9]. Hibiscus planting time substantially impacts the wet weight of the bush, the wet/dry weights of the boll, 1000 seed weight, total performance, and the harvested seed. The periods of planting on 9th March and 30th April caused a decrease in the weight of the bush and bolls [10]. The roselle planted on 15th April improved and yielded more leaves and branches, taller plants, and heavier leaf DW per plant compared to the 1st May, 15th May, and 1st June, respectively. In contrast, the earliest day of sowing (15th April) demonstrated greater values of roselle yield components with substantial variations compared to other dates [11]. Early dates of planting yielded the greatest number of branches, plant heights, dry and fresh weights of plants, seeds, and sepals, increased total carbohydrates percent, nutrient contents, anthocyanin, and acidity in sepals, as well as fixed oil in seeds [12]. The planting date shift from mid-May to mid-July resulted in a 60-percent drop in flower yield and a 58-percent decrease in the yield of calyxes [7].

On the other hand, water stress, a widespread environmental issue, negatively impacts crop yield, quality, and biomass production [13]. Plants respond with strategies like essential and auxiliary reactions to cope with temporary stress, but prolonged stress can negatively impact growth and yield [14–16]. Furthermore, saving irrigation water and improving crop yields are two related and important global issues [6]. In this concern, it has been stated that extending the period between drought and irrigation conditions led to a reduction in roselle components and yield but an increase in active calyces, including total phenols and vitamin C [17].

Irrigation management plays a critical role in regulating plant growth and productivity by influencing water availability, nutrient uptake, photosynthetic activity, and carbohydrate accumulation. Water deficit conditions can reduce plant height, stem diameter, biomass production, and photosynthetic efficiency, whereas adequate irrigation enhances resource utilization and promotes vegetative growth through improved acquisition of nutrients, water, and light interception [18]. Similar responses have been reported in roselle, where irrigation regimes significantly affected growth characteristics, yield components, and water use efficiency [19].

Moreover, Rah Khosravani et al. [18] and Seghatoleslami et al. [19] illustrated that irrigation water rates don’t significantly affect roselle plant growth but affect chlorophyll content in leaves. In addition, sowing dates affect calyx water use efficacy, seed oil content, antioxidant activity, calyx yield, and biological yield. In contrast, the interaction between irrigation periods and sowing date treatments did not significantly affect any trait [6]. El-Dissoky et al. [20] found that the increase in calyx yield for roselle is affected by irrigation frequency and mild drought stress. The results of Zand-Silakhoor et al. [6] highlighted that water stress has decreased the number of flowers and calyx since the flowering stage entails multiple processes prone to stress conditions. Additionally, roselle’s total anthocyanin content was significantly affected under mild and severe drought stress [6].

Even with the availability of several studies discussing the effects of sowing date or irrigation regimes on roselle performance, most studies have tested these factors independently. This gap restricts the ability to develop integrated strategies that simultaneously optimize yield and water productivity under water-limited conditions. It is hypothesized that examining sowing dates under different irrigation regimes can significantly influence roselle growth, yield, and maximize water productivity while ensuring acceptable yield under water-limited conditions. Water deficit triggers a complex network of physiological and biochemical responses in plants aimed at maintaining cellular homeostasis and protecting metabolic functions. Under drought conditions, plants frequently accumulate secondary metabolites such as anthocyanins, which act as antioxidants capable of scavenging reactive oxygen species (ROS) generated during stress. These compounds also contribute to photoprotection and stabilization of cellular membranes. In addition, drought stress often influences nutrient uptake, chlorophyll stability, and photosynthetic efficiency, ultimately affecting biomass accumulation and yield formation. Therefore, understanding the interaction between agronomic management practices, such as sowing date and irrigation regime, and plant physiological responses is essential for improving crop performance under water-limited environments [21–24]. Although several studies have evaluated the individual effects of planting date or irrigation management on roselle growth and yield, information regarding their interactive effects on physiological traits, secondary metabolite accumulation, and irrigation water productivity remains limited, particularly under semi-arid environments. Moreover, understanding how sowing date may modify plant responses to deficit irrigation is essential for developing climate-resilient production strategies under increasing water scarcity conditions. We hypothesized that the response of roselle to deficit irrigation depends on sowing date, and that an appropriate combination of planting time and irrigation regime may improve yield performance, physiological traits, and irrigation water productivity under water-limited conditions. Therefore, the present study was conducted to evaluate the combined effects of different sowing dates and irrigation regimes on growth, yield, anthocyanin accumulation, nutrient status, chlorophyll content, and irrigation water productivity of roselle under Egyptian field conditions. The study also aimed to identify the most suitable management strategy for maximizing productivity while improving water-use efficiency under limited water availability.

## Materials and methods

### Experimental site

Through the two summer seasons of 2021 and 2022, an experimental study was established in a split-plot design with three replicates, the sowing dates (T1; May 19, T2; June 19, and T3; July 19) as main plots and irrigation rates (I1; 100%, I2; 75%, and I3; 50% of crop evapotranspiration “ETc”) as sub-plots. The experiment was conducted at the AL-Busili Experimental Farm of the Central Laboratory for Agricultural Climate, Agricultural Research Center, Rashid City, Beheira Governorate, Egypt, 31°27’15” N, 30°23’23” E. The soil’s physical and chemical analysis (Table 1) was carried out by taking a sample of the soil at a 0–30 cm depth before planting, according to Cottenie et al. [25] and Kult et al. [26].

**Table 1:** Soil mechanical and chemical analysis of the experimental site

Sandy loam												
Soil texture												
Clay (%)		Silt (%)		Sand (%)	Organic matter (%)		CaCO_3_ (%)	Field capacity (%)		Wilting point (%)		Bulk density gcm^-3^
6.48		10.00		83.52	0.76		3.26	16.8		7.7		1.2
Available nutrients (mg kg^-1^ soil)				Soluble Cations (meq L^-1^)				Soluble Anions (meq L^-1^)			pH	EC (dS m^-1^)
N	P		K	Ca^2+^	Mg^2+^	Na^+^	K^+^	Cl^-^	HCO3^-^	SO4^2-^		
30.86	5.20		200	3.35	5.0	10.09	0.86	7.2	2.4	9.7	7.78	1.93

To convert meq L^-1^ to mmol L^-1^, divide by the valence of the ion

### Meteorological data

**Fig. 1:**
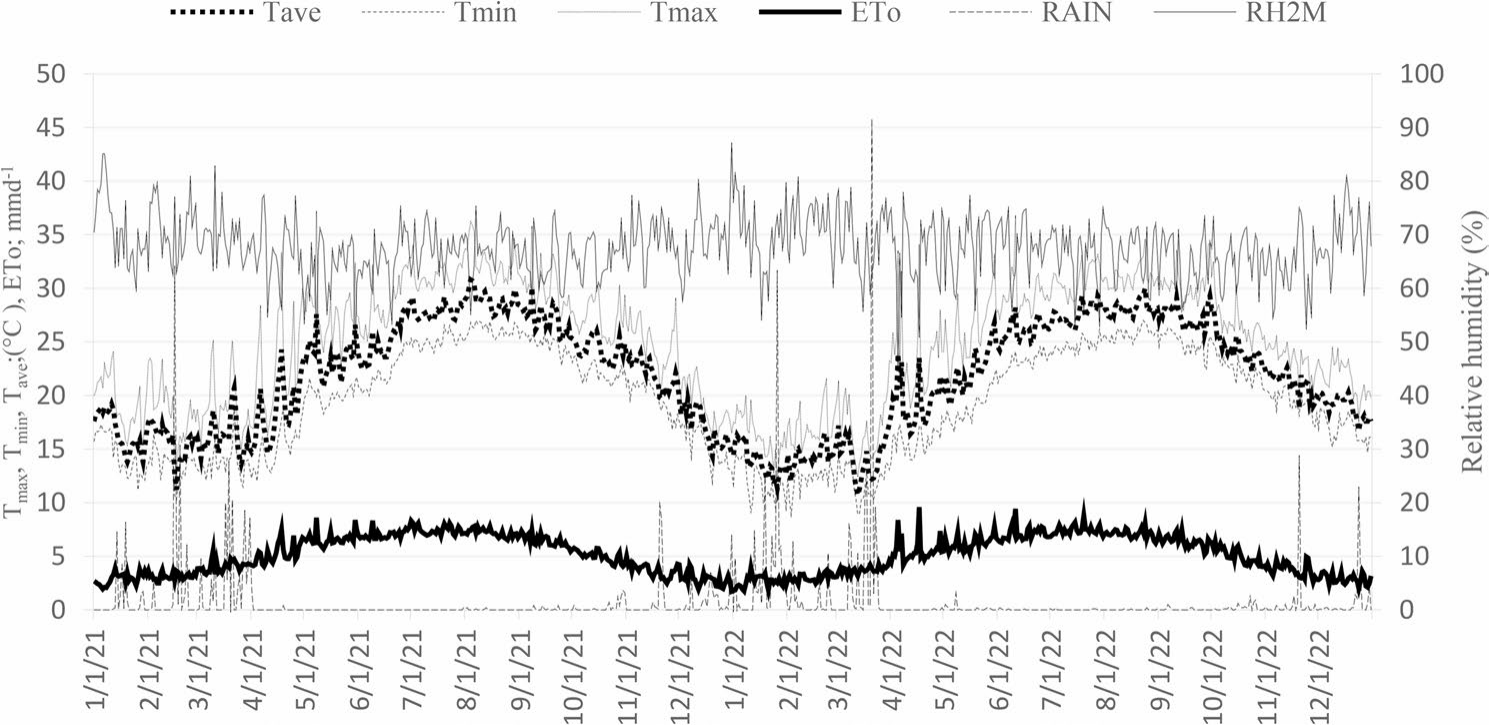
Weather data for the study area in 2021 and 2022, growing seasons

The daily meteorological values during the experimental period were gathered from the CLAC automated weather station located at the experimental site, as depicted in Fig. 1. The reference evapotranspiration (ET_o_, mm day^-1^) was determined using the method of Penman-Monteith (PM) [27].

### Field experiments

Inland preparation, the soil was well plowed, where compost as organic fertilizers at a rate of 48 m^3^ ha^-1^ and calcium super phosphate (15.5% P_2_O_5_) at 715 kg ha^-1^ were added. Roselle seeds (*H. sabdariffa* L. cv. Sabahia 17 dark) were provided by the Medicinal and Aromatic Plants Research Department, Horticulture Research Institute, Agricultural Research Center, Giza, Egypt. The experiment comprised nine treatments, and each experimental plot was 2 × 1.0 m (3 m^2^) and had two rows, 50 cm apart and 50 cm between the plant holes. Then, the treatments were replicated three times in a total of 27 plots, and each replicate encompassed ten plants. Seeds were sown on May 19, T_1_ (early), June 19, T_2_ (mid), and July 19, T_3_ (late) in both seasons. Three weeks after the seeds were sown, plants were thinned to one plant/hole. Irrigation treatments were initiated three weeks after sowing, and were applied at three day intervals through a drip irrigation system according to the designated irrigation regimes (I1; 100% ETc “regular”, I2; 75% ETc “mild”, and I3; 50% ETc “severe”), the plants received the recommended dosage of nitrogen, phosphorus, and potassium fertilizers, which were added at a rate of 475 kg ha^-1^ as ammonium sulfate (20.5% N), 360 kg ha^-1^ as potassium sulfate (48% K_2_O), and 60 L ha^-1^ as phosphoric acid (85% H_3_PO_4_) through fertigation. The conveying pipeline system consists of a 63 mm PVC main line connected to a 50.8 mm PVC sub-main line. It was a surface drip system with a 50-hp irrigation pump coupled to sand and screen filters. The sub-main line is connected to the 16 mm-diameter drip lateral lines. With built-in emitters with a 2 L h^-1^ discharge rate placed 0.3 m apart on the lateral lines, each 20 m long lateral line is separated 0.7 m apart on the sub-main. Small amounts of soluble water fertilizers were injected via a tank attached to the drip irrigation system. Other agricultural practices, including the use of insecticides, hoeing, and weeding, were all promptly applied to improve crop development according to the Ministry of Agriculture and Land Reclamation recommendations.

To minimize potential interference among irrigation treatments, adjacent experimental plots were separated by buffer zones of 1.0 m width. In addition, each irrigation treatment was supplied through an independent drip-irrigation lateral connected to a separate control valve, allowing precise regulation of the irrigation amount applied to each treatment. Irrigation was carefully monitored throughout the growing season, and the buffer areas were maintained without measurements to reduce any possible lateral movement of water between neighboring plots.

### Anthocyanin content

Anthocyanin content was determined following a spectrophotometric method. Dried roselle sepals (0.5 g) were extracted using acidified methanol (methanol: water: HCl, 79:20:1 v/v/v) and kept in darkness at 4 °C for 24 h. The extract was centrifuged at 5000 rpm for 10 min, and the absorbance of the supernatant was measured at 530 nm using a UV–visible spectrophotometer. Anthocyanin concentration was calculated using a calibration curve prepared with cyanidin-3-glucoside as the reference standard and expressed as mg cyanidin-3-glucoside equivalents per g dry weight (mg g⁻¹ DW) [28–31].

### Crop irrigation water calculation

Levels of irrigation were estimated, while manual valves were used to regulate irrigation for each experimental plot. Food and Agriculture Organization (FAO) Penman-Monteith (PM) procedure, the FAO 56 method was utilized to estimate the total quantity of irrigation water [27]. The first step entailed the calculation of reference evapotranspiration (ETo) as follows:

1 $${\boldsymbol{E}\boldsymbol{T}}_{\boldsymbol{o}}=\frac{\boldsymbol {0.408}\varDelta\:\left({\boldsymbol{R}}_{\boldsymbol{n}}-\boldsymbol{G}\right)+\boldsymbol{\gamma\:}\frac {\boldsymbol{900}}{\boldsymbol{T}+\boldsymbol{273}}{\boldsymbol{u}}_{2}({\boldsymbol{e}}_{\boldsymbol{s}}-{\boldsymbol{e}}_{\boldsymbol{a}})}{\varDelta\:+{\boldsymbol\gamma\:}(\boldsymbol1+\boldsymbol{0.34}{\boldsymbol{u}}_{\boldsymbol2})}$$

where: ET_o_ = Daily reference evapotranspiration [mm d^-1^]. R_n_ =Net radiation at the crop surface (MJ m^-2^ d^-1^). G = Soil heat flux density (MJ m^-2^ d^-1^), T = Mean daily air temperature at 2 m height (°C), U_2_ = Wind speed at 2 m height (m s^-1^), e_s_ = Saturation vapor pressure (kPa), e_a_ = Actual vapor pressure (kPa), Δ = The slope of the vapor pressure curve (kPa °C-1), γ = The psychometric constant (kPa °C^-1^).

The second step was to determine crop evapotranspiration (ETc) values according to Doorenbos and Pruitt [32].

2 $$ET_{c}=\;ET_\mathit 0 \times Kc \,\,\,\,\,\, mm\mathrm{.}d^{-{\mathit{1}}}$$

where: ET_o_ = Evapotranspiration rate from an excessive surface of green cover of uniform height (8 to 15 cm), entirely shading the ground, actively growing, with no water shortage, Kc = Crop coefficient, crop coefficient values were used (between 0.4 and 1.2).

Leaching requirements were determined according to Allen et al. [27] as follows.

3 $$\boldsymbol{LR}=\left(\frac{\boldsymbol{{E}{C}{i}{w}}}{\boldsymbol{{E}{C}{d}}}\right)\times\:\boldsymbol{100}\:\:\:{\%}$$

where: LR = leaching requirements, ECiw = Electrical conductivity of irrigation water (0.36 dS m^-1^), ECd = Electrical conductivity of drainage water 1.7 dS m^-1^– maize salinity threshold. Therefore, the LR of the current study was 21.17%.

Water requirements (WR) were determined based on the following equation:

4 $$\boldsymbol{WR}=\boldsymbol{ETc}\left( \boldsymbol1+\frac{\boldsymbol{LR}}{\boldsymbol{100}}\right)\,\,\,\,\,\,\,\,\,\,\,\boldsymbol{mm \mathrm{.} d^{-1}}$$

The irrigation requirement (IR) was determined as follows:

5 $$\boldsymbol{{I}{R}}=\frac{\boldsymbol{{W}{R}}\times\:\boldsymbol{4200}\times\:\boldsymbol{100}}{\boldsymbol{1000}\times\:\boldsymbol{{E}{a}}}\:\:\:\:\boldsymbol{{{m}}^{3}\mathrm{.}{{f}{e}{d}}^{-1}\mathrm{.}{{d}}^{-1}}$$

where: Ea = The irrigation system’s efficiency (assumed 85% of the total applied water).

The water flow meter for all treatments determined the total quantity of irrigation water. depicts the seasonal irrigation quantities for roselle under varying irrigation treatments at the AL-Busili site for the three sowing dates during the two seasons. The plants were irrigated with 2 L h^-1^ capacity drippers utilizing the fertigation technique.

### Irrigation water productivity (WP_I_)

The irrigation water productivity (kg m^− 3^) of roselle was calculated according to the equation accessible by Zhang [33] as follows:

6 $${WP}_{I}=\frac{Crop\:Yeild\:\left(kg\:{fed}^{-1}\right)}{Applied\:Water\:\left({m}^{3}\:{fed}^{-1}\right)}$$

### Recorded data

At the end of each season, roselle fruits were harvested on the 15th of November, 15th of December, and 15th of January for each sowing date. Ten plants were randomly taken from each plot, and plant height (cm), no. of branches per plant, no. of fruit per plant, the weight of the dry sepals (g plant^− 1^), dry weight of plant (g), stem diameter (cm), roots dry weight (g plant^− 1^), seed yield per plant (g), and fresh fruit yield (ton ha^− 1^) were determined. The available macronutrient percentage, including N, K, and P, was assessed in the roselle dry herb based on the methods defined by Nessler’s method [34, 35]. Total chlorophyll was determined in leaves using SPAD-502, Konica, Minolta.

### Statistical analysis

Data were subjected to analysis of variance (ANOVA) according to the procedures described by Gomez and Gomez (1984) [36]. Treatment means were compared using Fisher’s Least Significant Difference (LSD) test at *P* ≤ 0.05. Pearson correlation coefficients, principal component analysis (PCA), and hierarchical cluster analysis were performed following the procedures described by Hair et al. (2019) [37]. The data from both seasons showed similar distributions based on preliminary Levene’s tests for homogeneity of variance and Shapiro-Wilk tests for normality; as a result, the seasonal data were combined and subjected to a joint analysis. Results are expressed as the mean value of three replicates ± standard deviation (SD). Boxplots were developed to demonstrate the disparity between sowing date and irrigation rates, whereas Pearson correlation coefficients were utilized to assess the correlation among traits. In the R project (version 3.4.5), the ggplot2 package was utilized for drawing a boxplot. The relatively high Pearson correlation coefficients observed in this study may be partially attributed to the use of treatment means from the combined seasonal dataset. Consequently, the reported correlations reflect treatment-level associations among traits and should be interpreted with appropriate caution.

## Results

### Vegetative growth

The analysis of variance (ANOVA) illustrated in Table 2 showed that both sowing date and irrigation treatments treatments had a significant effect at 5% (*p* ≤ 0.05) on all studied parameters during the 2021 and 2022 seasons,. A marked (*p* ≤ 0.05) correlation was detected between the sowing dates and the irrigation treatments, revealing an association with plant height, branch number per plant, plant dry weight, root dry weight, and stem diameter only in 2^nd^ season. Plant dry weight (g plant^− 1^) and root dry weight (g plant^− 1^) were significantly affected by the interaction between sowing date and irrigation rates in both seasons. However, plant height (cm), branch number per plant, and sepals’ dry weight (g plant^− 1^) were only affected in the 1st season. While the number of fruits per plant and stem diameter (mm) were only affected in the 2^nd^ season.

**Fig. 2:**
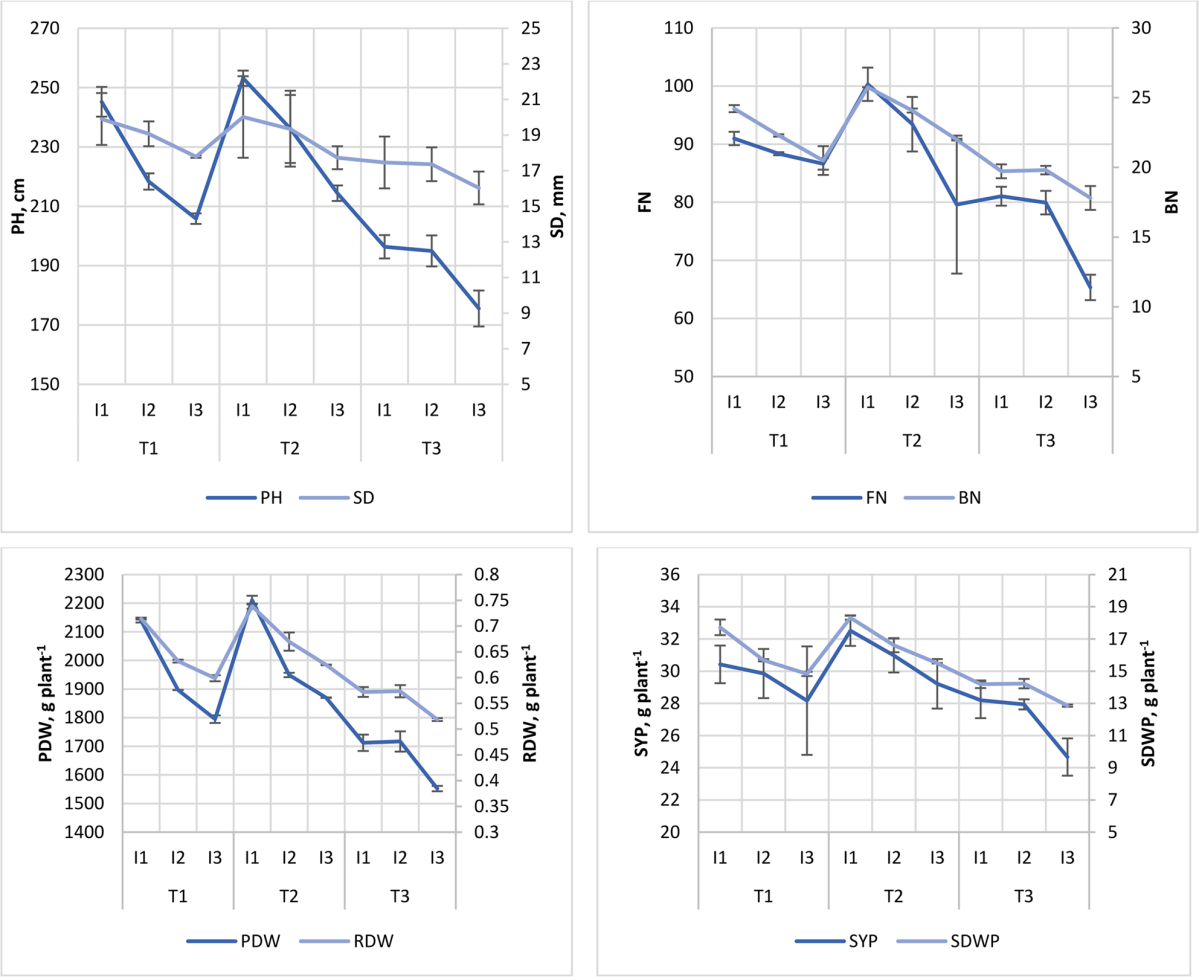
A combined analysis of two seasons for roselle vegetative growth as affected by varying sowing dates and irrigation rates. Where: T1 = 19^th^ May, T2 = 19^th^ June, T3 = 19^th^ July, I_1_= 100% ETc, I_2_= 75% ETc, I_3_= 50% ETc. Plant height (PH), Stem diameter (SD), Number of fruits per plant (FN), Number of branches per plant (BN), Plant dry weight (PDW), Roots dry weight (RDW), Seed yield per plant (SYP), Sepals dry weight per plant (SDWP). The error bar means ± SD (*n* = 3)

Moreover, as seen in Fig. 2, the mean data of both seasons revealed that (T2) with (I1) had the highest value of plant height (PH, 253.17 cm), stem diameter (SD, 20.01), fruit number per plant (FN, 100.30), branch number per plant (BN, 25.74), plant dry weight (PDW, 2210.89 g plant^− 1^), root dry weight (RDW, 0.74 g plant^− 1^), seed yield (SYP, 32.51 g plant^− 1^), and sepals (SDWP, 18.32 g plant^− 1^). In contrast, the late sowing date (T3) combined with severe water stress (I3; 50% ETc) yielded the lowest values for these parameters.

**Table 2:** Analysis of variance results for different traits of roselle under varying sowing dates and irrigation rates

Source of variance	Plant height (cm)		Branch Number per plant		Number of fruits per plant		Plant dry weight (g plant^− 1^)	
	2021	2022	2021	2022	2021	2022	2021	2022
Sowing date (T)	***	**	***	***	*	***	***	***
Irrigation rates (I)	***	***	***	***	**	***	***	***
T X I	**	ns	**	ns	ns	***	***	***
CV	2.180	3.088	2.745	3.634	8.997	3.503	1.783	1.67
R^2^	0.987	0.960	0.976	0.954	0.841	0.956	0.990	0.986
RMSE	4.721	6.627	0.565	0.836	7.452	3.058	33.408	31.28

	Stem diameter (mm)		Roots dry weight (g plant^− 1^)		Sepals dry weight (g plant^− 1^)		Seed yield (g plant^− 1^)	
Sowing date (T)	ns	**	***	***	***	***	*	ns
Irrigation rates (I)	ns	***	***	***	***	***	***	**
T X I	ns	*	**	***	***	ns	ns	ns
CV	9.819	2.776	2.364	1.77	1.79	3.60	2.80	6.58
R^2^	0.709	0.967	0.984	0.984	0.984	0.963	0.955	0.791
RMSE	1.770	0.515	0.015	0.011	0.277	0.562	0.811	1.929

significance at 5% (*p*≤ 0.05), R2 coefficient of determination

*RMSE* root mean square error, *CV* coefficient of variation

Statistical significance is indicated as follows: ns = not significant; * = *p* < 0.05; = *p *< 0.01; *** = *p* < 0.001

### Fruits yield (FW, ton ha^-1^)

**Fig. 3:**
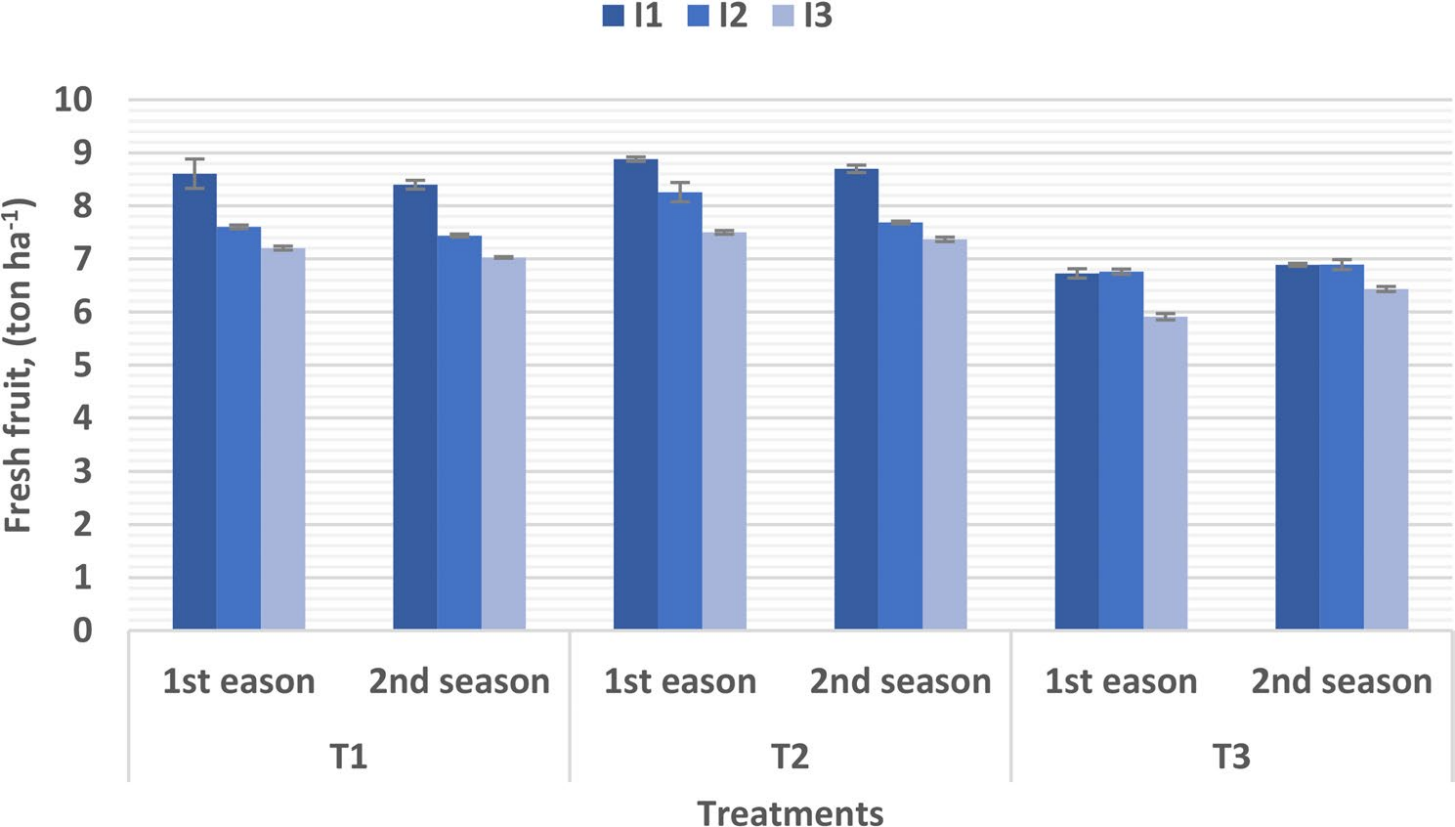
A combined analysis of two seasons for roselle fruit yield (FW, ton ha^− 1^) as affected by varying sowing dates and irrigation rates. Where: T1 = 19^th^ May, T2 = 19^th^ June, T3 = 19^th^ July, I_1_= 100% ETc, I_2_= 75% ETc, I_3_= 50% ETc. The error bar means ± SD (*n* = 3)

In the first season, under well-watered conditions (I1;100% ETc), delaying the sowing date from 19 May (T1) to 19 June (T2) increased the roselle fresh fruit yield from 8.62 ton ha^-1^ to 8.88 ton ha^-1^, representing an increase of 3.08%. However, further delaying sowing to 19 July (T3) significantly decreased fresh fruit yield to 6.74 ton ha^-1^, corresponding to a 21.84% decrease relative to T2 (Fig. 3). Similarly, under severe water stress (I3;50% ETc), delaying the sowing date from 19 May to 19 June increased the roselle fresh yield per ton by 3.97%, whereas a further delay to 19 July decreased the fresh fruit yield by 17.95%. Likewise, the second season showed the same trend as shown in the first season (Fig. 3).

### Anthocyanin content

A non-significant difference in anthocyanin content was observed across sowing dates (Table 3). Across both seasons, I2 and I3 resulted in relative increases in anthocyanin content ranging from 11% to 14% over 100% ETc, as shown in Table 3. Furthermore, combining sowing date and irrigation levels proved highly significant in effect, where T2 with I2 consistently yielded the highest anthocyanin concentrations, peaking at 52.69 mg g^-1^ F.W. across the 2^nd^ season.

**Table 3:** Anthocyanin content (mg g^-1^ d.w.) in roselle sepals as affected by sowing date and irrigation rates

Treatments	Sowing date (T1)							
Irrigation rates (I)	Season 2021				Season 2022			
	T1	T2	T3	Mean	T1	T2	T3	Mean
I1	45.04 ± 1.6	42.02 ± 1.4	33.06 ± 3.3	40.04b	48.40 ± 5.3	45.63 ± 1.8	36.60 ± 2.7	43.54 b
I2	44.12 ± 2.1	47.59 ± 1.2	43.45 ± 1.2	45.058a	48.99 ± 3.9	52.69 ± 4.5	46.96 ± 1.5	49.55 a
I3	45.45 ± 3.7	45.40 ± 0.7	46.58 ± 3.	45.814a	45.03 ± 1.6	48.72 ± 5.6	51.32 ± 2.9	48.35 a
Mean	44.87 a	43.63 a	42.42 a		47.48 a	49.02 a	44.96 a	
LSD_0.05_	T = 4.43		I = 1.82		T = 4.94		I = 3.53	
	T x I = 3.14^***^				T x I = 6.11^**^			

Different lowercase letters indicate statistically significant differences between treatments (*p*≤ 0.05), as revealed by the least significant difference (Fisher’s LSD) test. Where. T1= 19^th^ May, T2= 19^th^ June, T3=19^th^ July, I_1_= 100% ETc, I_2_= 75% ETc, I_3_= 50% ETc. Statistical significance is indicated as follows: ns = not significant; * = *p* < 0.05; = *p* < 0.01; *** = *p* < 0.001

### Macronutrient content

**Fig. 4:**
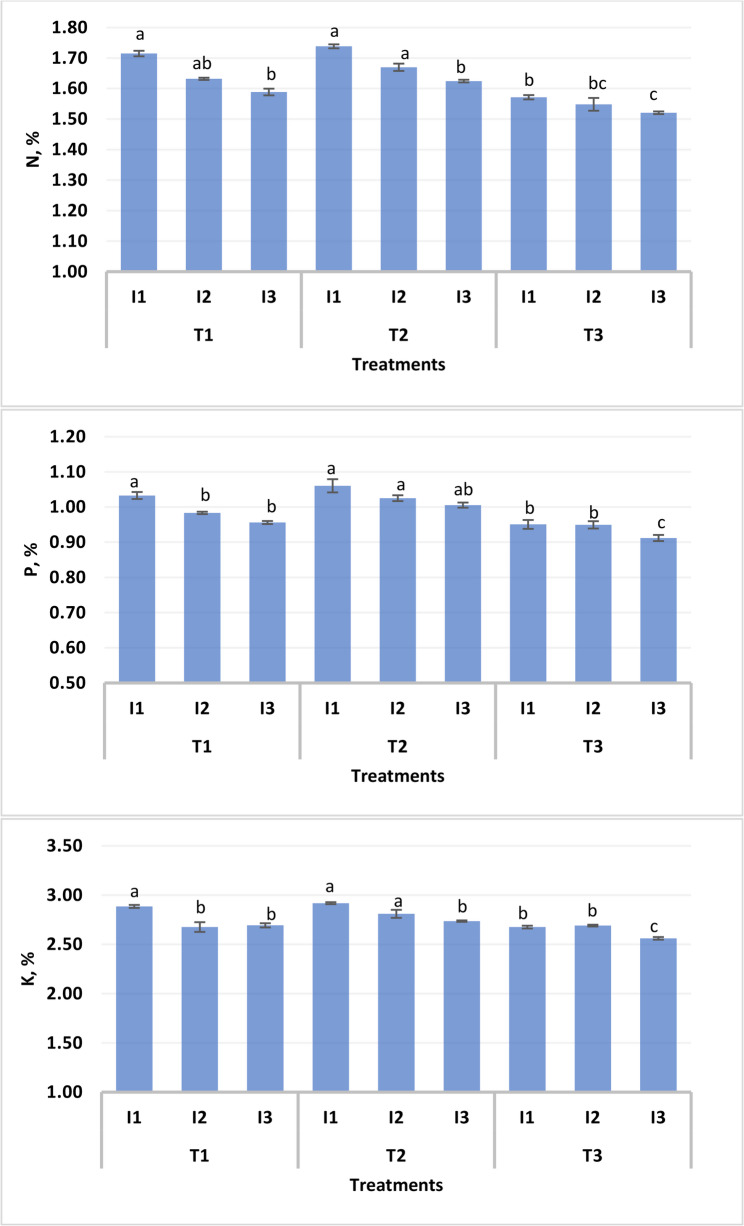
A combined analysis of two seasons for the available macronutrients content (%) in roselle leaves as affected by sowing date and irrigation rates. Where: T1 = 19^th^ May, T2 = 19^th^ June, T3 = 19^th^ July, I_1_= 100% ETc, I_2_= 75% ETc, I_3_= 50% ETc. Different letters above bars indicate significantly across treatments (LSD test, *p* ≤ 0.05). The error bar means ± SD (*n* = 3)

Concerning the interactive impact of the sowing dates and various irrigation rates on the available macronutrient content in leaves of roselle, the mean data of both seasons in Fig. 4 reflected that the irrigation level was at 100% ETc under the sowing date on the 19^th^ of May (T1) and 19^th^ of June (T2) and demonstrated the superiority of the N%, P%, and K% in leaves as compared with other treatments. Moreover, as shown in Fig. 4, no significant differences were registered in the N%, P%, and K% between I1 and I2 during T2 of the sowing date.

### Total chlorophyll

**Fig. 5:**
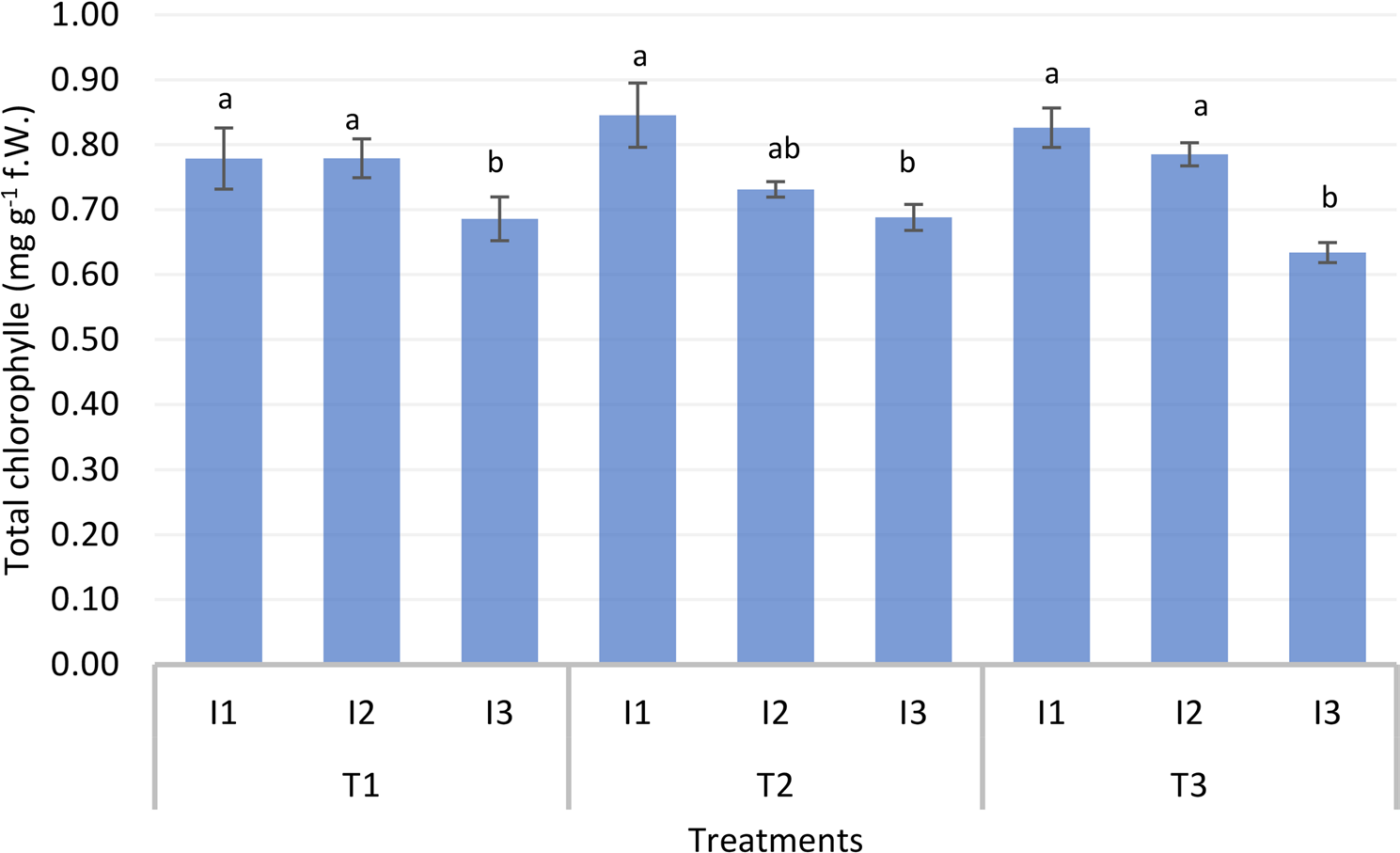
A combined analysis of two seasons for total chlorophyll (mg g^− 1^ f.w.) in roselle leaves as affected by sowing date and irrigation rates. Where: T1 = 19^th^ May, T2 = 19^th^ June, T3 = 19^th^ July, I_1_= 100% ETc, I_2_= 75% ETc, I_3_= 50% ETc. Different letters above bars indicate significantly across treatments (LSD test, *p* ≤ 0.05). The error bar means ± SD (*n* = 3)

The mean effects of sowing date and irrigation rates on total chlorophyll (mg g^-1^ f.w.) are presented in Fig. 5. Generally, there was a non-significant difference in the total chlorophyll content at the I1 (100% ETc) and I2 (75% ETc) under the dates of T1 and T3. On the other hand, the results presented that the adoption of sowing date (T2) increased total chlorophyll content (0.85 mg g^-1^ f.w.) under I1 treatment, although this increase was significantly equal to the application of T1 + I1 (0.87 mg g^-1^ f.w.) and T3 + I1. (0.83 mg g^-1^ f.w.).

### Correlation between studied traits

**Fig. 6:**
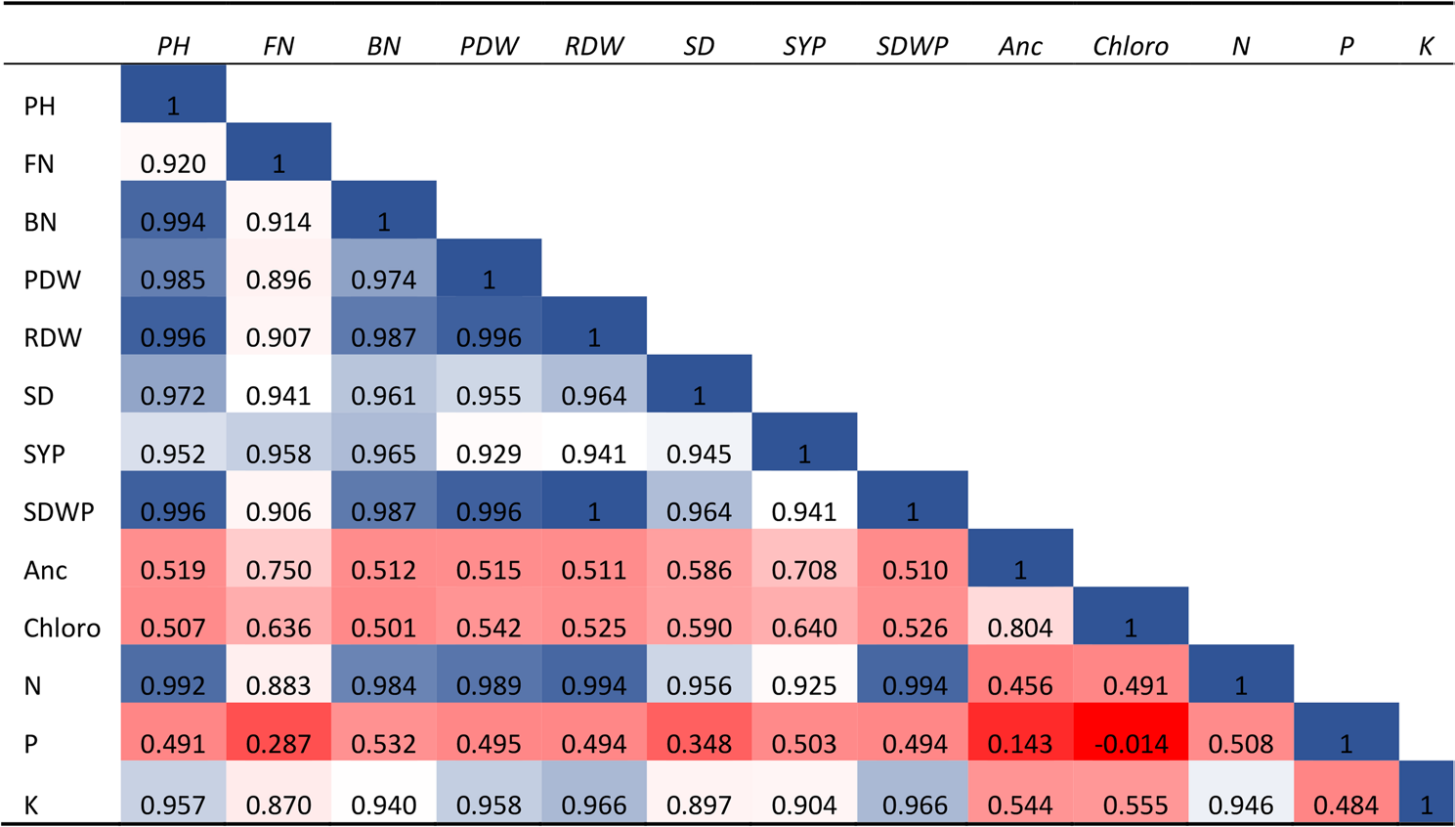
Pearson’s correlation coefficients for seven attributes examined under varying sowing dates and irrigation rates (Combined analysis of two successive seasons of 2021 and 2022). Plant height (PH); Number of fruits per plant (FN), Number of branches per plant (BN), Plant dry weight (PDW), Roots dry weight (RDW), Stem diameter (SD), Seed yield per plant (SYP), sepals dry weight per plant (SDWP); Anthocyanin content (Anc), chlorophyll content (Chloro), Nitrogen content (N), Phosphor content (P), and Potassium content (K). Colors indicate the strength and direction of the relationships. Blue represents positive correlations, while red represents negative correlations

Pearson’s correlation coefficients for different attributes examined under varying sowing dates and irrigation rates are presented in Fig. 6. Data revealed that the association between root dry weight and plant dry weight was substantially positive. The plant height (PH) revealed a strong positive correlation with plant dry weight (PDW), root dry weight (RDW), sepals dry weight per plant (SDWP), and nitrogen content (N). Likewise, branch number per plant (BN) expressed a positive correlation with PDW, RDW, stem diameter (SD), seed yield per plant (SYP), and SDWP. Also, the sepals’ dry weight per plant had a positive correlation with nitrogen content. Moreover, anthocyanin (Anc) and chlorophyll (Chloro) content exhibited a strong positive association with growth and yield parameters, including PH, FN, BN, PDW, RDW, SD, SYP, and SDWP. Notably, anthocyanin presented a particularly strong positive correlation with the number of fruits per plant (*r* = 0.750) and seed yield (*r* = 0.708), while chlorophyll exhibited its strongest correlation with seed yield (*r* = 0.640) and the number of fruits (*r* = 0.636). Determining the correlation between yield traits is necessary as it directly contributes to enhancing yield traits. The direct selection of these attributes might enhance the selection efficiency of yield.

### The correlation between irrigation rates and sowing date

**Fig. 7:**
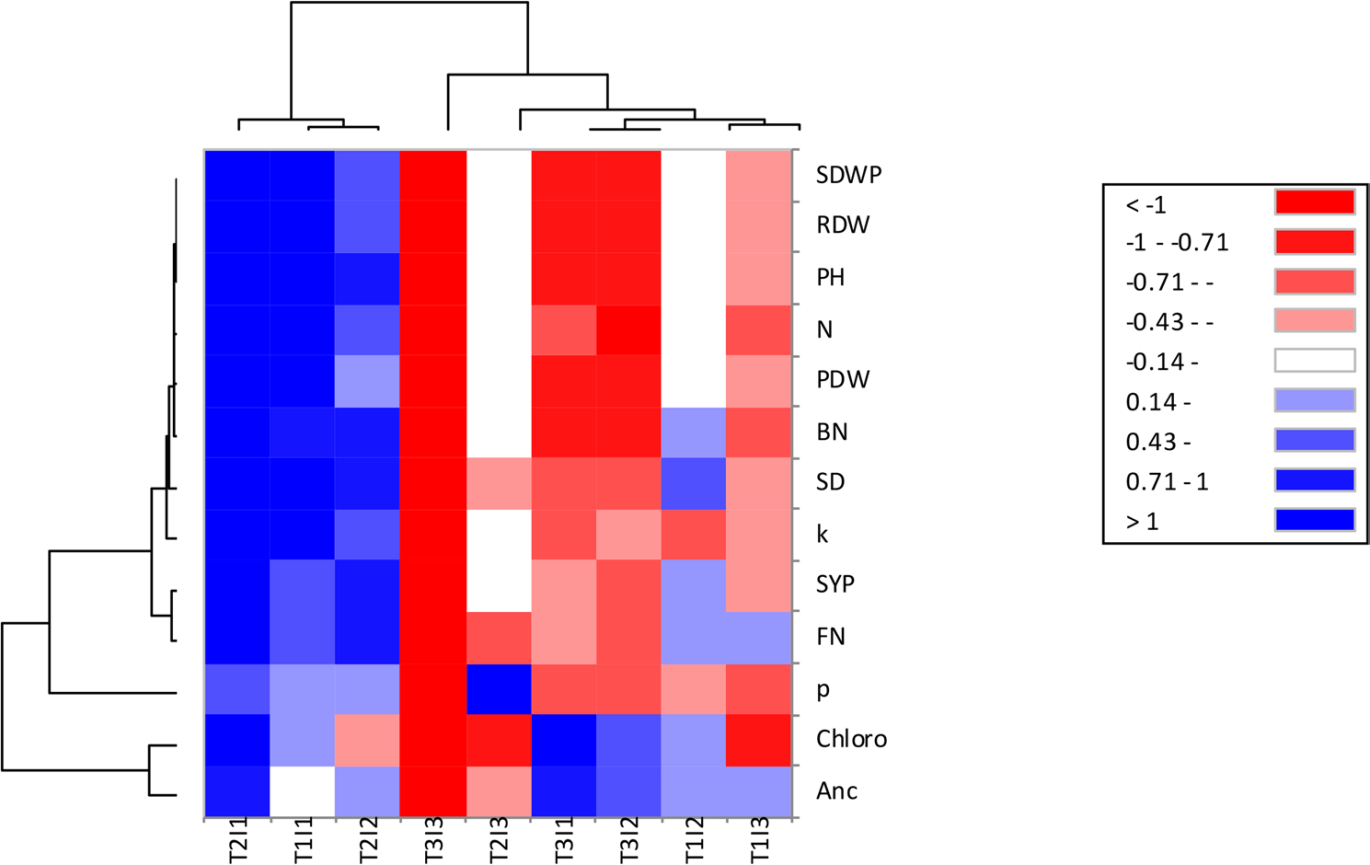
Clustering analysis of the interrelationships between examined traits, sowing date, and irrigation rates treatments. In the ballots, the hierarchical clustering analysis with the Euclidean distance using the principal component scores and Ward’s technique as the process of linkage was used. Plant height (PH); Number of fruits per plant (FN), Number of branches per plant (BN), Plant dry weight (PDW), Roots dry weight (RDW), Stem diameter (SD), Seed yield per plant (SYP), sepals dry weight per plant (SDWP); Anthocyanin content (Anc), chlorophyll content (Chloro), Nitrogen content (N), Phosphor content (P), and Potassium content (K). Where: T_1_= 19^th^ May, T_2_= 19^th^ June, T_3_=19^th^ July, I_1_= 100% ETc, I_2_= 75% ETc, I_3_= 50% ETc

The hierarchical clustering effectively identified the link between combinations of sowing dates and irrigation rates (12 combinations) based on their yield and growth performance parameters (Fig. 7). Two main clusters were characterized concerning the association between sowing dates and irrigation rates. The combination treatments of A (T2 + I1), B (T1 + I1), and C (T2 + I2) generated the first cluster. B and C treatments were the closest sub-clusters. Treatment A yielded the greatest values for most traits in this group. For treatment B, most traits, except SYP, FN, P, Chloro, and Anc, demonstrated high performance. Conversely, treatment C demonstrated the highest positive impacts on PH, BN, SD, SYP, and FN, followed by SDWP, RDW, N, and K, whereas chloro content was negatively impacted by this treatment. The second cluster consisted of treatments D (T3 + I3), E (T2 + I3), F (T3 + I1), G (T3 + I2), H (T1 + I2), and I (T1 + I3), with treatments F and G or H and I being the closest sub-clusters. The combinations of sowing dates and irrigation rates in the second cluster demonstrated an opposite pattern to the first cluster combinations. In the second cluster, most studied traits were adversely affected. While the anthocyanin content followed a similar downward trend in the most severe stress combinations, it remained relatively higher in specific deficit irrigation treatments (I2 and I3) compared to full irrigation, which is consistent with its role as a stress-response metabolite.

### Principal component analysis

**Fig. 8:**
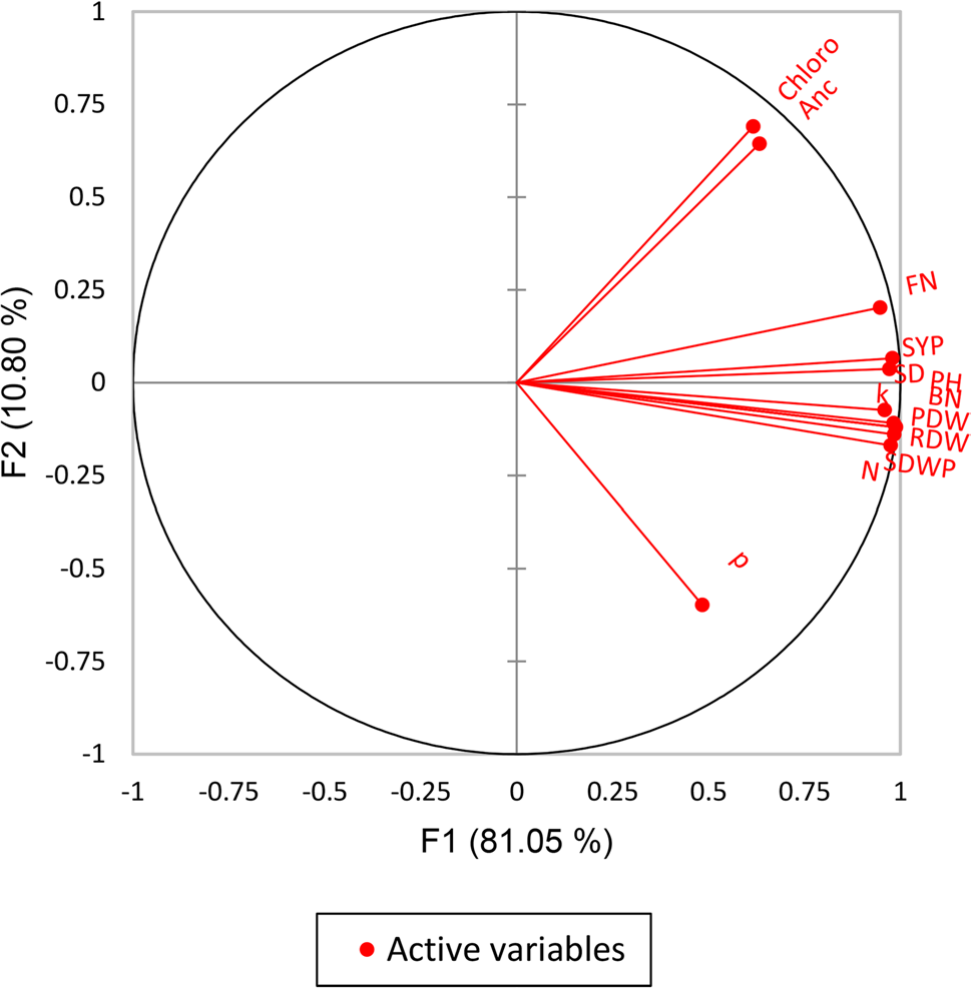
PCA analysis of the correlations between traits based on the sowing dates and levels of irrigation within two years. Diagrams are defined by the first two axes of the PCA of the different variables (*n* = 3): Axis1 (illustrating 81.05% of variance) and Axis2 (illustrating 10.80% of variance). Where Plant height (PH); Number of fruits per plant (FN), Number of branches per plant (BN), Plant dry weight (PDW), Roots dry weight (RDW), Stem diameter (SD), Seed yield per plant (SYP), sepals dry weight per plant (SDWP); Anthocyanin content (Anc), chlorophyll content (Chloro), Nitrogen content (N), Phosphor content (P), and Potassium content (K)

PCA was carried out to determine the association between the examined treatments and traits (Fig. 8). The two PCs are responsible for 91.85% of the variance. F1 seemed to be linked to the T1I2, T2I1, T2I2, and T1I1 on the positive side to T3I1, T3I2, T1I3, T2I3, and T3I3 on the negative side, which illustrates 81.05% of the variability, whereas F2 contributed to 10.8% of the variation (Fig. 8). The angles between trait vectors demonstrated the relationship between the investigated characteristics. Vectors with large angles (about 180 degrees) exhibit a negative correlation, while contiguous vectors exhibit a significant positive correlation. A high positive correlation was found between anthocyanin and chlorophyll content and all of its characteristics. Due to the simplicity of their measurement, the closeness of FN, SYP, and SD to anthocyanin content indicates their significance in indirect selection (Fig. 8).

### Applied irrigation water

**Fig. 9:**
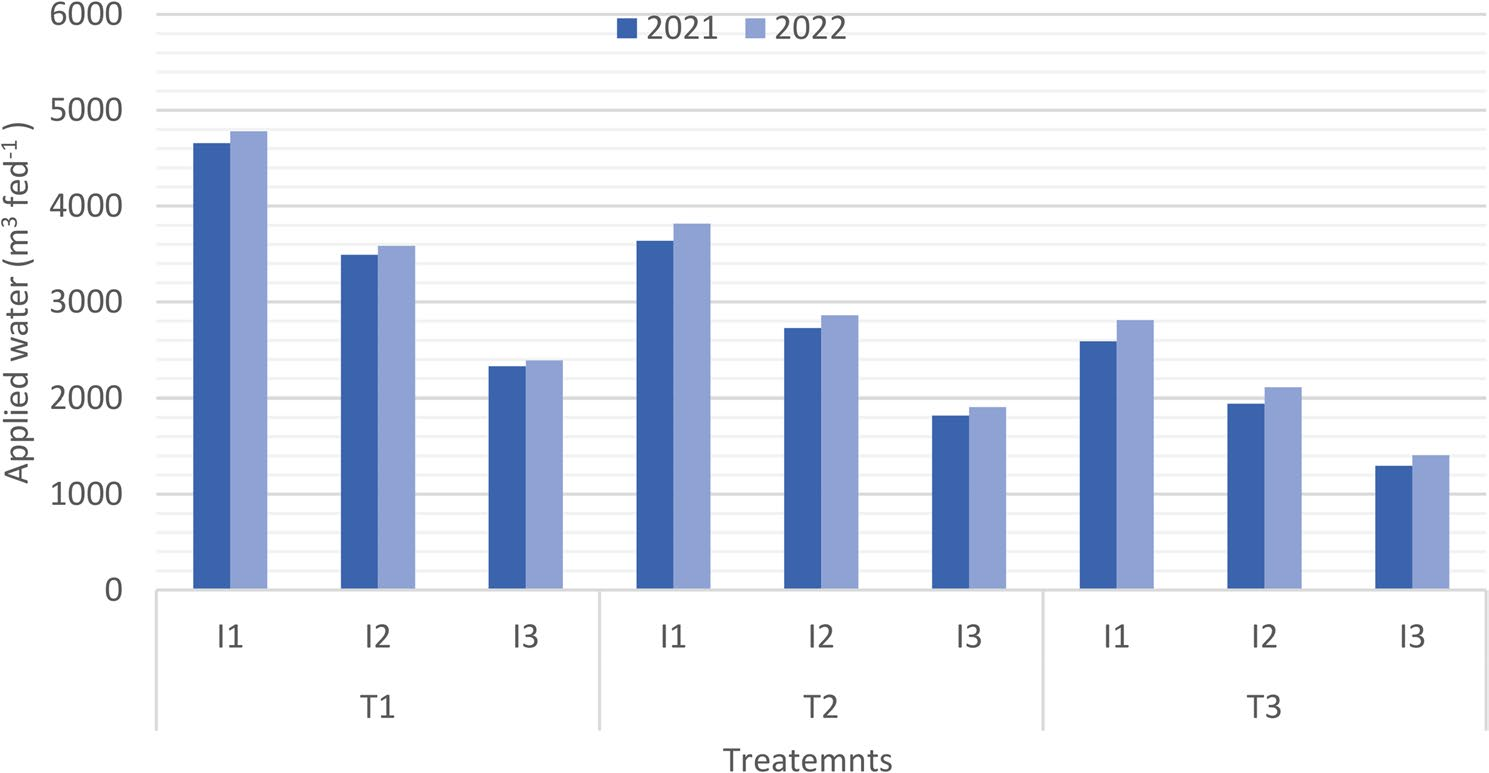
Seasonal irrigation quantities of roselle under various sowing dates at the AL-Busili site

The applied irrigation water varied among different sowing dates and irrigation rates (Fig. 9). The applied irrigation water values for roselle fluctuated between 2997.6 and 11088.9 m^3^ ha^-1^ in 2021 and 3347.3 to 11379 m^3^ ha^-1^ in 2022. The early-sown plants at T1 recorded the highest irrigation demand as the crop coefficient reached its maximum values during July and August, when Tmin, Tmax, and ETo values were maximum (Fig. 1). While delaying sowing dates at T3 treatments registered the lowest applied irrigation water when periods of peak climatic factors coincided with low crop coefficient values. As a result of changes in the meteorological components, the results also revealed that the applied water had higher values in the second season than in the first season.

### Irrigation water productivity (WP_I_, kg m^-3^)

**Fig. 10:**
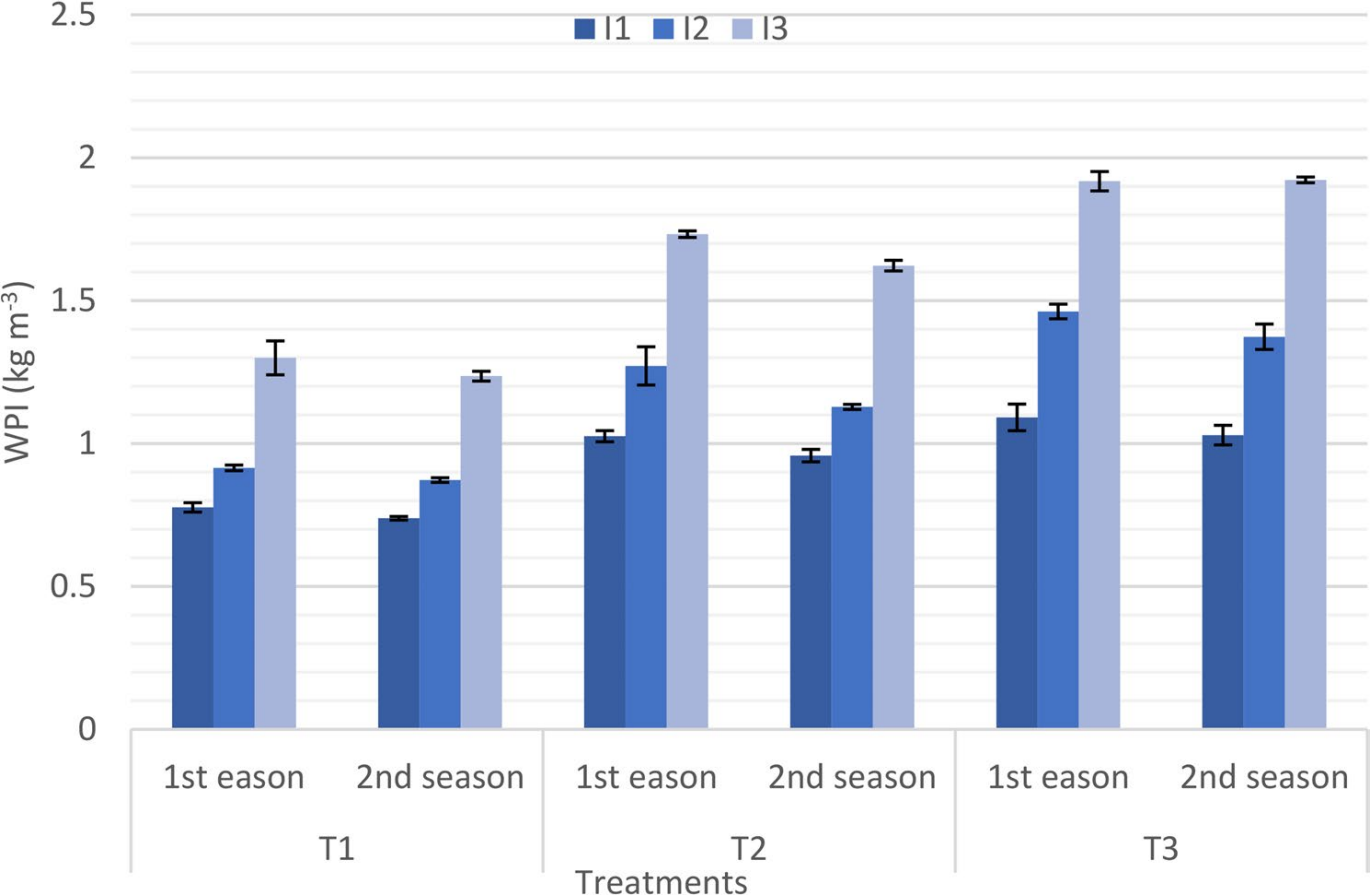
Irrigation water productivity (kg m^− 3^) of both growing seasons of roselle as impacted by different sowing dates and irrigation rates. Where: T1 = 19^th^ May, T2 = 19^th^ June, T3 = 19^th^ July, I_1_= 100% ETc, I_2_= 75% ETc, I_3_= 50% ETc

In both seasons, the highest irrigation water productivity for fresh sepals (1.917 and 1.922 kg m^− 3^, in the first and second seasons, respectively) was obtained under severe water stress (I3; 50% ETc) combined with the late sowing date (T3; 19^th^ July). These values were 32.22% and 9.66% higher than those recorded under the early (T1; 19^th^ May) and mild (T2; 19^th^ June) sowing dates, respectively, in the first season, and 36.50% and 15.60% higher in the second season under the same condition of irrigation (50% ETc). Conversely, the lowest IWP values (0.776 and 0.738 kg m^− 3^) were obtained under full irrigation (I1; 100% ETc) with the early sowing date (T1; 19^th^ May) in the first and second seasons, respectively (Fig. 10). These results indicate that severe water stress maximized irrigation water productivity, resulting in the highest fresh sepal yield per unit of irrigation water applied, despite a reduction in absolute yield.

## Discussion

Under climate change scenarios characterized by increasing water scarcity and temperature variability, understanding plant physiological responses to water deficit becomes increasingly important. The results of the present study suggest that roselle plants can activate protective metabolic responses under moderate water limitation, including enhanced accumulation of stress-related metabolites. Such responses may contribute to maintaining productivity in semi-arid environments and highlight the potential of integrating physiological insights with agronomic management strategies.

Deficit irrigation may have stimulated adaptive responses that contributed to improved tolerance to water deficit conditions. Similar responses have been associated with enhanced antioxidant activity in previous studies; however, antioxidant enzymes were not directly measured in the present study. These adaptive mechanisms help maintain cell turgor, protect membrane integrity, and reduce oxidative damage caused by drought-induced reactive oxygen species (ROS). The observed responses in roselle plants under different irrigation regimes in the present study likely reflect such physiological adjustments that enable plants to maintain growth and productivity under limited water availability [22].

The current experiment was conducted to determine the optimal sowing date and irrigation rates for improved roselle productivity and water use efficiency. This result may be due to the effect of the day and night temperature, daylight intensity, and photoperiods. In addition, these results are probably because the sowing dates in June led to the high growing period and the irrigation rates received during the growing periods. An adequate supply of moisture would eventually boost the growth rate as well as timely maturity, hence a higher productivity of the roselle. These results are in agreement with El-Bakhshwan and El-Kouny [38], who indicated that the plant height (cm), number of branches per plant, and number of fruit decreased in the roselle sown in July compared with the roselle sown in June. El-Bakhshwan and El-Kouny [38] found that all parameters of hibiscus growth were significantly increased as affected by irrigation water level (1000 m^3^) and the sowing period in June. Moreover, our findings align with Barzgaran’s [39] results on *H. Sabdariffa*, who confirmed that planting time has a greater influence on the performance of other agronomic features. The outcome indicates that delaying the planting of this crop would be optimal (19^th^ June). The date of planting on 19^th^ June corresponded to the greatest values of the examined parameters. Khattak et al. [12] discovered the same pattern in roselle plants.

The superior growth and yield observed under the 14-day irrigation interval may be associated with improved water availability during critical developmental stages, which likely supported cell expansion, nutrient uptake, and assimilate translocation. These responses could have contributed to maintaining vegetative growth and reproductive development under favorable moisture conditions. Similar trends have been reported in roselle and other crops subjected to optimized irrigation management [15, 22].

It was observed that the maximum increase in anthocyanin content was observed when the sowing date 19 of June was adopted with the implementation of I_2_ (75%ETc) and I_3_ (50% ETc). The accumulation of anthocyanins under reduced irrigation levels observed in the present study may represent an adaptive protective mechanism against drought-induced oxidative stress. Anthocyanins are widely recognized as potent antioxidants capable of scavenging reactive oxygen species generated during environmental stress. In addition to their antioxidant function, these pigments may contribute to photoprotection by absorbing excess radiation and protecting photosynthetic tissues from photooxidative damage. Therefore, the observed responses likely reflect physiological and biochemical adjustments to water deficit conditions, although the specific metabolic pathways involved were not investigated in the present study [21, 22, 36]. The higher yield observed under the 14-day irrigation interval could be associated with improved physiological performance and resource utilization under adequate water availability. However, photosynthetic parameters were not directly assessed in this study, as reported in numerous articles [37, 40].

The increase in anthocyanin accumulation under water-deficit conditions may represent an adaptive response to environmental stress. Anthocyanins have been reported to participate in plant protective mechanisms by contributing to oxidative stress mitigation and cellular protection. Therefore, the enhanced anthocyanin concentration observed in the present study may reflect a physiological adjustment aimed at maintaining plant performance under limited water availability [23, 24].

This demonstrates a way to control light absorption and lessen damage caused by photooxidation. According to reports, the observed increase in anthocyanin content may indicate a protective response to water deficit conditions. Anthocyanins have been reported to participate in stress adaptation and antioxidant-related processes in plants, although these mechanisms were not directly evaluated in the present study, hence reducing photo-oxidative damage in leaves [41]. Moreover, the impact that drought stress has on TAC is consistent with the findings of [42, 43] on roselle, which reported that enhanced secondary metabolism has been linked to improvements in the plants’ qualitative traits, like anthocyanins, under simultaneous drought stress. These results are in agreement with [6, 9, 44].

Environmental stresses such as drought are known to stimulate the biosynthesis of secondary metabolites through the activation of stress-related metabolic pathways. The enhanced accumulation of anthocyanins under reduced irrigation regimes in the present experiment may therefore reflect a metabolic shift toward protective secondary compounds. Such responses are commonly associated with improved stress tolerance, as secondary metabolites often function as antioxidants, osmoprotectants, and signaling molecules involved in plant defense mechanisms [22, 41].

On the other hand, the results showed that the accumulation of N, P, K, and total chlorophyll in roselle leaves reached its highest values when T2 (19 June) was combined with I_1_ (100% ETc) and I_2_ (75% ETc), while the lowest values were detected when roselle seeds were sown on 19^th^ of July with I_3_ (50% ETc). The authors postulate that extreme heat and evaporation conditions begin to impair plant growth on July 19. Plants respond to these conditions by increasing their uptake and storage of K as a defensive mechanism. K contains several qualities that can enhance temperature and plant water status beneath water. The results are in line with [6, 12, 45] on *Hibiscus sabdariffa*, and [46–48] on *Foeniculum vulgare.*

Maintenance of chlorophyll content under water deficit conditions is considered an important indicator of drought tolerance in many crop species. Drought stress often accelerates chlorophyll degradation due to oxidative damage and impaired nutrient uptake, ultimately leading to reduced photosynthetic capacity. However, plants capable of maintaining higher chlorophyll levels under moderate water stress may sustain more stable photosynthetic activity and carbon assimilation. The variations in chlorophyll content observed in this study suggest that roselle plants may possess physiological mechanisms that partially protect the photosynthetic apparatus under moderate water limitation [48–50].

The reduction in chlorophyll content under prolonged irrigation intervals may indicate impairment of photosynthetic apparatus stability under water-deficit conditions. Water stress has frequently been associated with reduced chlorophyll biosynthesis and accelerated pigment degradation, which may limit light-harvesting efficiency and ultimately constrain biomass production [21].

Although the maximum water productivity of irrigation was obtained under severe water deficit (50% ETc), this result was associated with reductions in roselle yield compared with full irrigation. Hence, there is a typical conflict in maximizing the water productivity or roselle yield. From the agronomic point of view, the combination of full irrigation (100% ETc) and June sowing date maximized yield components and biomass of the roselle plant, while severe deficit irrigation (50% ETc) improved the water productivity of the roselle plant. Therefore, both treatments can be considered in optimizing approaches. For sustainable production systems, the optimal irrigation technique should be selected depending on the objective of the system, especially the limitation of available water resources. In arid and semi-arid conditions, improving the water productivity of deficit irrigation may maximize limited irrigation water usage. However, where ensuring crop yield is a priority, moderate water deficit (75% ETc) may reserve a balanced compromise between irrigation water productivity and yield performance [15, 33].

Despite the valuable findings obtained in this study, the experiment was conducted at a single location under specific environmental and soil conditions over two consecutive growing seasons. Therefore, caution should be exercised when extrapolating the results to other agroecological regions with different climatic and edaphic conditions. In addition, although several agronomic, physiological, and quality-related traits were evaluated, the study did not directly assess drought-related physiological mechanisms such as photosynthetic performance, antioxidant enzyme activities, osmotic adjustment, or reactive oxygen species accumulation. Future research should include multi-location and long-term field trials, together with detailed physiological and biochemical investigations, to further validate the observed responses and improve the understanding of roselle adaptation to deficit irrigation under diverse environmental conditions.

Although the observed responses suggest the involvement of physiological and biochemical adaptation mechanisms under deficit irrigation, the present study did not directly evaluate antioxidant enzyme activities, reactive oxygen species accumulation, photosynthetic performance, or other stress-related physiological parameters. Therefore, interpretations regarding these mechanisms should be considered indicative rather than conclusive and warrant further investigation in future studies. Collectively, the results of this study demonstrate that roselle plants exhibit multiple adaptive responses to water deficit conditions, including modulation of photosynthetic pigments, accumulation of antioxidant secondary metabolites, and adjustments in growth-related traits. These responses reflect coordinated physiological and metabolic strategies that allow plants to balance growth and stress protection under limited water availability. Understanding these mechanisms is essential for developing agronomic strategies that enhance crop resilience under increasing drought frequency associated with climate change.

## Conclusions

This study demonstrate that optimizing sowing date and irrigation management can significantly influence roselle productivity and physiological responses under water-limited conditions. The results indicated that sowing roselle on 19 June combined with 100%ETc maximized its vegetative growth and fruit yield. However, while 50% ETc significantly reduces individual plant biomass, it achieves the highest water productivity when sowed on 19 July. Moderate to severe water deficit promoted anthocyanin accumulation, which may represent an adaptive biochemical mechanism contributing to stress tolerance. These results highlight the importance of integrating agronomic practices with plant physiological responses to enhance crop performance under climate-induced water scarcity. In conclusion, late sowing in Egypt can enhance water productivity for roselle fruits despite limited water availability. However, further multi-year and multi-location studies are essential to verify these findings across different climatic scenarios.

## Data Availability

All data generated or analyzed during this study are included in this published article.

## References

[1] Mohamed R, Fernandez J, Pineda M, Aguilar M. Roselle (Hibiscus sabdariffa) seed oil is a rich source of γ-tocopherol. J Food Sci. 2007;72:S207–11. 17995816 10.1111/j.1750-3841.2007.00285.x

[2] Ismail A, Ikram EHK, Nazri HSM. Roselle (Hibiscus sabdariffa L.) seeds nutritional composition protein quality and health benefits. Food. 2008;2:1–16.

[3] Hassan FAS. Response of Hibiscus sabdariffa L. plant to some biofertilization treatments. Annals Agric Sci. 2009;54(2):437–46.

[4] Xu H, Wang X, Zhao C, Zhang X. Responses of ecosystem water use efficiency to meteorological drought under different biomes and drought magnitudes in northern China. Agric Meteorol. 2019;278:107660.

[5] Keshavarz Mirzamohammadi H, Modarres-Sanavy SAM, Sefidkon F, Mokhtassi-Bidgoli A, Mirjalili MH. Irrigation and fertilizer treatments affecting rosmarinic acid accumulation, total phenolic content, antioxidant potential and correlation between them in peppermint (Mentha piperita L). Irrig Sci. 2021;39:671–83.

[6] Zand-Silakhoor A, Madani H, Sharifabad HH, Mahmoudi M, Nourmohammadi G. Influence of different irrigation regimes and planting times on the quality and quantity of calyx, seed oil content and water use efficiency of roselle (Hibiscus sabdariffa L). Grasas Aceites. 2022;73:e472–472.

[7] Ghayour M, Taherian M, Baghban S, Khavari S. Effect of early planting dates and different treatments of seed priming on germination and seedling establishment of roselle (Hibiscus sabdariffa). Iran J Seed Res. 2020;6:95–109.

[8] Butler TJ, Evers GW, Hussey MA, Ringer LJ. Flowering in crimson clover as affected by planting date. Crop Sci. 2002;42:242–7. 11756281 10.2135/cropsci2002.2420

[9] Attia EM, Khater RM. Effect of different planting dates and organic fertilizers treatments on growth and yield of Hibiscus sabdariffa L. plants. Egypt J Desert Res. 2015;65:153–70.

[10] Behzadi M, Emamipour Y, Koduri MR, Sardoei AS. The effect of planting time on performance of Roselle (Hibiscus sabdariffa) to use in urban green space. J Mddle east Appl Sci Technol. 2014;24:156–9.

[11] Ali HKM, Awad AE, Abdelkader MA. Improving growth and yield of Roselle (Hibiscus sabdariffa L.) plants by using tyrosine and glutamine acids under different sowing dates. Zagazig J Agricultural Res. 2020;47:1165–74.

[12] Khattak AM, Sajid M, Sarwar HZ, Rab A, Ahmad M, Khan MA. Effect of Sowing Time and Plant Density on the Growth and Production of Roselle (Hibiscus sabdariffa). Int J Agric Biol. 2016;18:1219–24. 10.17957/IJAB/15.0215.

[13] Laskari M, Menexes G, Kalfas I, Gatzolis I, Dordas C. Water stress effects on the morphological, physiological characteristics of maize (Zea mays L.), and on environmental cost. Agronomy. 2022;12:2386.

[14] Seleiman MF, Al-Suhaibani N, Ali N, Akmal M, Alotaibi M, Refay Y, et al. Drought stress impacts on plants and different approaches to alleviate its adverse effects. Plants. 2021;10:259. 33525688 10.3390/plants10020259 PMC7911879

[15] Haggag Wafaa M, Tawfik MM, Abouziena HF, Abd El Wahed MSA, Ali RR. Enhancing Wheat Production Under Arid Climate Stresses Using Bio-Elicitors. Gesunde Pflanzen. 2017;69:149–58.

[16] He M, He C-Q, Ding N-Z. Abiotic stresses: general defenses of land plants and chances for engineering multistress tolerance. Front Plant Sci. 2018;9:1771. 30581446 10.3389/fpls.2018.01771 PMC6292871

[17] Hewidy M, Elsayed MLM, Sultan E. Water schedule of roselle (Hibiscus sabdariffa L.) under organic fertilization. Egypt J Hortic. 2018;45:53–64.

[18] Rah Khosravani AT, Mansourifar C, Modarres Sanavy SAM, Asilan KS, Keshavarz H. Effects of sowing date on physiological characteristics, yield and yield components for different maize (Zea mays L.) hybrids. Not Sci Biol. 2017;9:143–7.

[19] tk 2Seghatoleslami MJ, Mousavi SG, Barzgaran T. Effect of irrigation and planting date on morpho-physiological traits and yield of Roselle (Hibiscus sabdariffa). J. Anim. Plant Sci. 2013;23(1):256–60.

[20] El-Dissoky R, Attia AM, Awad AM. Managing Roselle Plant (Hibiscus sabdariffa L.) Requirements of Fertilizers and Irrigation Grown under Upper Egypt Conditions. J Soil Sci Agricultural Eng. 2020;11:693–700.

[21] Li Z, Ahammed GJ. Plant stress response and adaptation via anthocyanins: A review. Plant Stress. 2023;10:100230.

[22] Jan R, Asif S, Asaf S, Lubna, Khan Z, Kim K-M. Unveiling the protective role of anthocyanin in rice: insights into drought-induced oxidative stress and metabolic regulation. Front Plant Sci. 2024;15:1397817. 38863532 10.3389/fpls.2024.1397817 PMC11165195

[23] Dabravolski SA, Isayenkov SV. The role of anthocyanins in plant tolerance to drought and salt stresses. Plants. 2023;12:2558. 37447119 10.3390/plants12132558 PMC10346810

[24] Permatasari NA, Pöhnl T, Neugart S. Impact of Drought, Salinity, and Their Combination on Growth, Mineral Content, and Plant Secondary Metabolites of Tomatoes (Solanum lycopersicum L). Physiol Plant. 2026;178:e70725. 41467623 10.1111/ppl.70725 PMC12751611

[25] Cottenie A, Verloo M, Kiekens L, Velghe G, Camerlynck R. Chemical analysis of plants and soils. Lab Agroch State Univ Gent Belgium. 1982;63:44–5.

[26] Klute A, Page AL. Methods of soil analysis. 2nd Edition, American Society of Agronomy and Soil Science Society of America, Madison, USA. 1986;9.

[27] Allen RG, Pereira LS, Raes D, Smith M. Crop evapotranspiration-Guidelines for computing crop water requirements-FAO Irrigation and drainage paper 56. Volume 300. Rome: Fao; 1998. p. D05109.

[28] Manjula G, Krishna H, Mushrif SK, Manjunatha Reddy T, Shankarappa T. Standardization of anthocyanin extraction from Roselle (Hibiscus sabdariffa L.) calyces for edible colour. Pharma Innov J. 2022;11:1337–42.

[29] Gartaula C, Karki DB. Optimization of Extraction of Anthocyanins from Roselle (Hibiscus sabdariffa var. sabdariffa) in Aqueous Medium. J Food Sci Technol Nepal. 2010;6:69–72.

[30] Mohamed RK, Gibriel AY, Rasmy NMH, Abu-Salem FM. Extraction of anthocyanin pigments from Hibiscus sabdariffa L. and evaluation of their antioxidant activity. Middle East J Appl Sci. 2016;6:856–86.

[31] Mazibuko DM, Maskey S, Kurashina K, Okazawa H, Oshima H, Kato T, et al. Effects of Biochar on Growth, Response to Water Stress, and Post-Stress Recovery in Underutilized Vegetable Hibiscus sabdariffa from Malawi. Crops. 2025;5:13.

[32] Doorenbos J, Pruitt WO. Crop water requirements. FAO irrigation and drainage paper 24. Volume 144. Rome: Land and Water Development Division, FAO; 1977.

[33] Zhang C-H. Compound decision theory and empirical Bayes methods. Ann Stat. 2003;379–90.

[34] Chapman HD, Pratt PE, Parker F. Methods of analysis for soils, plants and waters. Univ Calif Div Agric Sci Priced Pub. 1978;4034:50–169.

[35] Jackson ML. Soil Chemical Analysis,(2nd Indian Print) Prentice-Hall of India Pvt. Ltd New Delhi. 1973;38:336.

[36] Hinojosa-Gómez J, San Martín-Hernández C, Heredia JB, León-Félix J, Osuna-Enciso T, Muy-Rangel MD. Anthocyanin induction by drought stress in the calyx of roselle cultivars. Molecules. 2020;25:1555. 32231098 10.3390/molecules25071555 PMC7180819

[37] Cirillo V, D’Amelia V, Esposito M, Amitrano C, Carillo P, Carputo D, et al. Anthocyanins are key regulators of drought stress tolerance in tobacco. Biology (Basel). 2021;10:139. 33578910 10.3390/biology10020139 PMC7916658

[38] El-Bakhshwan MH, El-Kouny H. Impact of irrigation intervals, planting time and density on hibiscus yield. Misr J Agricultural Eng. 2018;35:485–500.

[39] Barzgaran T. Effects of irrigation and planting date on agronomic traits and yield of roselle. Unpublished M Sc thesis, Dept of Agriculture, Islamic Azad Univ Birjand Branch, Iran 111p. 2011.

[40] An J, Zhang X, Bi S, You C, Wang X, Hao Y. The ERF transcription factor MdERF38 promotes drought stress-induced anthocyanin biosynthesis in apple. Plant J. 2020;101:573–89. 10.1111/tpj.14555. 31571281 10.1111/tpj.14555

[41] Wang J, Chen X, Lu X, Yang J, Huang H, Zhou X-R, et al. Anthocyanins syntheses genes and abiotic stress: A review in cotton. Ind Crops Prod. 2025;237:122165.

[42] Fallahi H-R, Ghorbany M, Aghhavani-Shajari M, Samadzadeh A, Asadian AH. Qualitative response of roselle to planting methods, humic acid application, mycorrhizal inoculation and irrigation management. J Crop Improv. 2017;31:192–208.

[43] Hinojosa-Gómez J, San Martín-Hernández C, Heredia JB, León-Félix J, Osuna-Enciso T, Muy-Rangel MD. Anthocyanin induction by drought stress in the calyx of roselle cultivars. Molecules. 2020;25:1555. 32231098 10.3390/molecules25071555 PMC7180819

[44] Khalil SE, Yousef RMM. Study the effect of irrigation water regime and fertilizers on growth, yield and some fruit quality of Hibiscus sabdariffa L. Int J Adv Res (Indore). 2014;2:738–50.

[45] Sanjeet Bagari SB, Singh PP, Naruka IS, Rathore SS, Shaktawat RPS. Effect of date of sowing and nitrogen levels on growth, yield and quality of fennel. Indian J. Hortic. 2010;67(04):518–24.

[46] El-Khayat ASM, Gouda HAH. Effect of sowing date and potassium fertilization on growth, yield and chemical composition of Foeniculum vulgare Mill plants. Annals of Agric Sci Moshtohor. Egypt, Fac of Agric Zagazig Univ. 2005;43:1245–69.

[47] El-Wahab MAA, Mehasen HRA. Effect of locations and sowing date on (Foeniculum vulgare Mill.) Indian fennel type under Upper Egypt conditions. J Appl Sci Res. 2009;677–85.

[48] Monteoliva MI, Guzzo MC, Posada GA. Breeding for drought tolerance by monitoring chlorophyll content. Gene Technol. 2021;10:10–35248.

[49] Shin YK, Bhandari SR, Jo JS, Song JW, Lee JG. Effect of drought stress on chlorophyll fluorescence parameters, phytochemical contents, and antioxidant activities in lettuce seedlings. Horticulturae. 2021;7:238.

[50] Scalon SPQ, Dresch D, Foresti AC, Santos CC, Pereira ZV. Chlorophyll a fluorescence as an indicator of water stress in Calophyllum brasiliense. Not Bot Horti Agrobot Cluj Napoca. 2020;48:210–20.

